# Extrinsic Adenomyosis Is Associated With Postoperative Recurrence of Ovarian Endometrioma

**DOI:** 10.3389/fmed.2021.815628

**Published:** 2022-01-12

**Authors:** Man Sun, Ping Xu, Gen Zou, Jianzhang Wang, Libo Zhu, Xinmei Zhang

**Affiliations:** The Department of Gynecology, Women's Hospital, Zhejiang University School of Medicine, Hangzhou, China

**Keywords:** ovarian endometrioma, recurrence, extrinsic adenomyosis, DIE, primary fertility

## Abstract

**Objective:** To determine whether endometrioma recurrence is closely related to the presence of extrinsic adenomyosis, which was demonstrated by magnetic resonance imaging (MRI).

**Design:** Observational crosssectional study involving patients with the recurrence of ovarian endometrioma (OMA). Correlations of endometrioma recurrence and adenomyosis subtypes shown by MRI were analyzed.

**Method:** Between January 2018 and December 2020, a total of 233 patients with recurrence of OMA after ovarian cystectomy were administered for surgery at our institution. All patients were divided into subtype II (Group A), subtype I+IV (Group B), and nonadenomyosis (Group C) groups at preoperative MRI imaging. The correlations of endometrioma recurrence with clinical features, imaging appearance, and surgical findings were retrospectively analyzed.

**Results:** We found 112 (48.07%) patients of endometrioma recurrence combined with subtype II adenomyosis, 8 (3.43%) subtype I adenomyosis, 47 (20.17%) subtype IV adenomyosis, 66 (28.32%) nonadenomyosis. The mean time of OMA recurrence (44.28 ± 8.37, vs. 63.96 ± 10.28, vs. 69.36 ± 9.34 mon), rate of pain symptoms (85.71, vs. 69.10, vs. 18.18%), and primary infertility (31.25, vs. 14.55, vs. 10.77%) were higher in Group A. Uterine volume (257.37± 42.61, vs. 203.14 ± 33.52, vs. 100.85 ± 26.67 cm^3^), and mean OMA size (4.97 ± 2.25, vs. 4.36 ± 2.38, vs. 4.46 ± 2.70 cm) were significantly larger in Group A. The rate of DIE (83.93, vs. 45.45, vs. 40.91%), the number of DIE (3.6 ± 1.8 vs. 2.3 ± 1.5 vs. 2.2 ± 1.3), the mean total revised American Society for Reproductive Medicine score (rASRM, 103.14 ± 23.89 vs. 74.23 ± 16.72 vs. 36.51 ± 14.23) were significantly higher in Group A. After a multiple logistic regression analysis, extrinsic adenomyosis (OR 2.5, 95% CI 1.2–3.4), DIE lesions (OR 2.1, 95% CI 1.4–2.8), and primary infertility (OR 1.8, 95% CI 1.3–4.3) were significantly associated with early recurrence (in 3-year) of OMA.

**Conclusions:** Extrinsic adenomyosis was associated with postoperative recurrence of OMA. In addition, a pathogenic link between extrinsic adenomyosis and pelvic endometriosis needs to be clarified.

## Introduction

Endometriosis is a chronic benign gynecological disease, usually presenting with pelvic pain and infertility (1). Based on the location of the lesion, endometriosis is divided into peritoneal, ovarian endometrioma (OMA), or deep infiltrating endometriosis (DIE) (2). Ovarian endometriosis is the most common type. Surgery results in benefit in pain relief and fertility outcomes. However, young women are more likely to have postoperative endometriosis recurrence, and the recurrence rate is higher (3). The recurrence rate of OMA following surgical treatment is up to 50%, even in those who are receiving postoperative hormonal suppression intervention (4). Adenomyosis is frequently combined with endometriosis even in recurrent endometriosis (5).

Adenomyosis is defined by the presence of endometrial tissue within the myometrium. The subtype of adenomyosis was often presented according to different configurations in the myometrium: diffuse adenomyosis and focal adenomyosis (6). Magnetic resonance imaging (MRI) is widely used in diagnosing adenomyosis with high accuracy. According to Kishi's criteria, adenomyosis demonstrated by MRI appears to consist of 4 distinct subtypes of different causes: subtype I adenomyosis (intrinsic), subtype II adenomyosis (extrinsic), subtype III adenomyosis (intramural), and subtype IV adenomyosis (indeterminate) (7). The clinical presentations of different adenomyosis are also heterogeneous, especially in pelvic pain, menstrual bleeding, and infertility (8). It is now widely recognized that there is a strong clinical relationship between endometriosis and adenomyosis according to their respective phenotypes (9). In MRI radiologic diagnosis, focal extrinsic adenomyosis more frequently occurred in OMA patients and was significantly associated with DIE phenotype (10). In molecular biology, Ber-EP4 (epithelial cell marker) and CD10 (stromal cell marker) of extrinsic adenomyosis were similar with coexistent DIE lesions (11). In contrast, the pattern of gland and stromal cells in the cases with intrinsic adenomyosis were similar to the endometrium (12). It is still unknown whether extrinsic adenomyosis should be considered as a variant of adenomyosis or a disease that originated from pelvis OMA, which subsequently invades into the outer myometrium (13). The association between recurrent endometriosis and adenomyosis has still not been fully elucidated.

Unfortunately, determinants of clinical characteristics for the recurrence of OMA are not well known. Ovarian endometriosis, coexisting with adenomyosis during the first surgery, was with a higher rate of recurrence (14). Stage III-IV endometriosis with adenomyosis has a lower pregnancy rate (15). Therefore, endometriosis should be considered in postoperative management. Up to the present, there is still a lack of high-quality data evaluating different adenomyosis combined with OMA and whether extrinsic adenomyosis is involved in the recurrence of endometriosis.

The purpose of this work is to investigate the relationship between extrinsic adenomyosis demonstrated by MRI and the recurrence of OMA. We compared the clinical features, imaging appearance (MRI and ultrasound), and surgical findings in extrinsic adenomyosis (subtype II) with other phenotypes of adenomyosis. We evaluated risk factors of early recurrence of OMA related to adenomyosis. We hope to provide a comprehensive understanding of the relationship between extrinsic adenomyosis and recurrent endometriosis.

## Materials and Methods

This is a retrospective crosssectional study conducted at the Department of General Gynecology, Women' Hospital, School of Medicine, Zhejiang University. We recruited all the patients who underwent laparoscopic cystectomy and were pathologically diagnosed with recurrent OMA from January 2018 to December 2020. This study was approved by the Ethics committee of Women's Hospital School of Medicine, Zhejiang University (ethics approval No. IRB-20210343-R). The inclusion criteria consists of the following conditions: (1) reccurent endometriosis who received surgical treatment; (2) ultrasonography was conducted to determine endometrioma recurrence from at least 6 months after surgery or recurrence of clinical symptoms including pelvic pain (dysmenorrhea, dyspareunia, or noncyclic pelvic pain); (3) histopathological diagnosed with EM after operation; (4) complete clinical and pathological data; (5) premenopausal. The exclusion criteria were as follows: (1) age <20 or age >45 years; (2) having undergone bilateral oophorectomy or hysterectomy; (3) women with infectious disease (e.g., sexually transmitted disease, tubo-ovarian abscess) or cancer.

**Figure 1:**
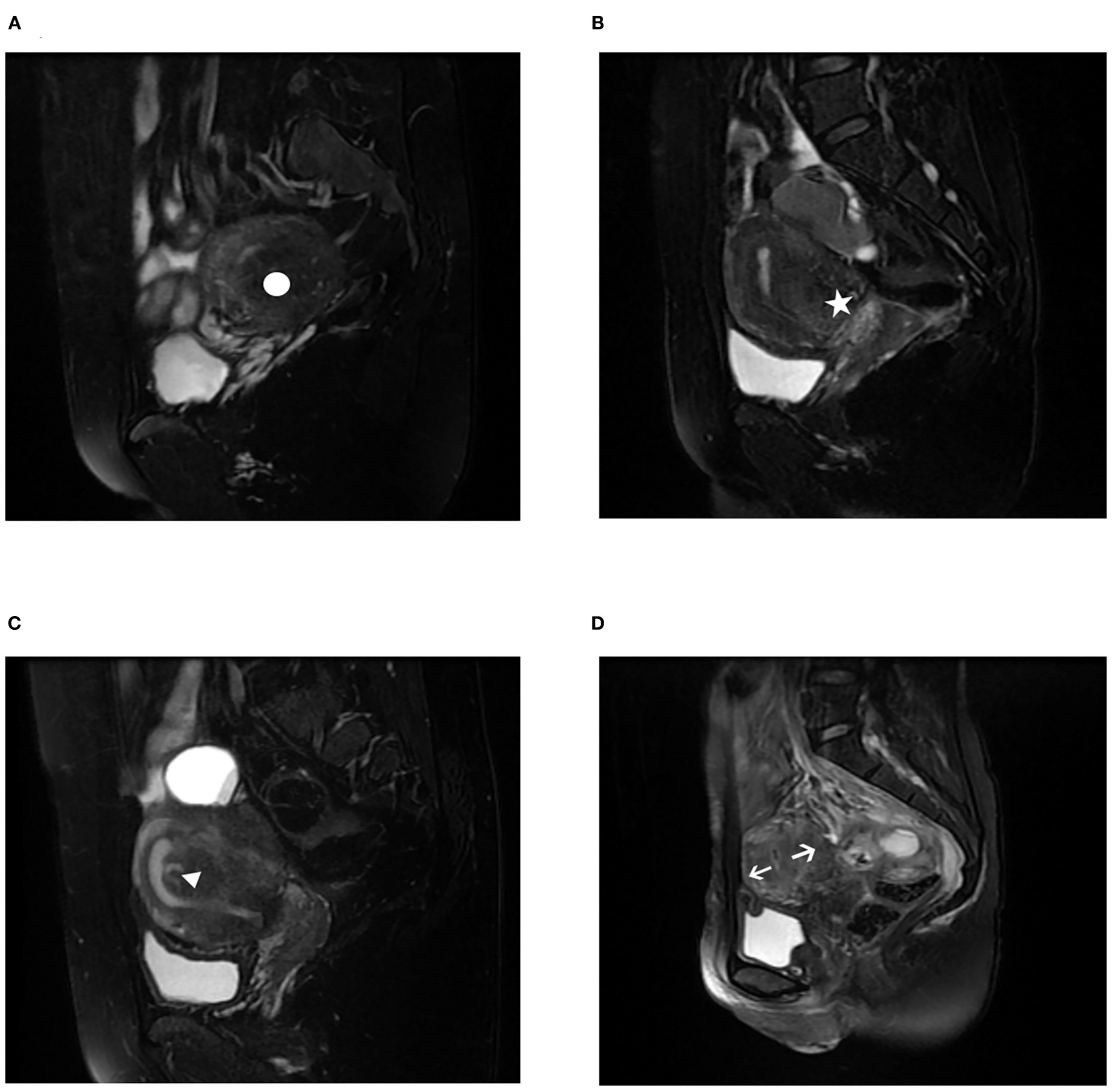
Magnetic resonance imaging (MRI) characteristics of different subtypes of adenomyosis on T2-weighted acquisitions. **(A)** subtype I (intrinsic adenomyosis) adenomyosis, white circle; **(B)** subtype II (extrinsic adenomyosis) adenomyosis, white star; **(C)** subtype III (intramural adenomyosis) adenomyosis, white triangle; **(D)** subtype IV (indeterminate adenomyosis) adenomyosis, white arrow. In the 233 recurrence of ovarian endometriosis, we found it was combined with 112 (48.07%) subtype II adenomyosis, 8 (3.43%) subtype I adenomyosis, 47 (20.17%) subtype IV adenomyosis.

In this study, adenomyosis was demonstrated based on imaging methods, such as MRI examination. All the patients had a preoperative pelvic MRI examination on T2-weighted acquisitions that allowed adenomyosis to be diagnosed. The diagnosis and categorization of adenomyosis by MRI were established when the agreement of the common diagnosis and subtype by the three radiologists was reached. Subtype I (intrinsic) originates from direct endometrial invasion and affects the junctional zone of the uterus. Subtype II (extrinsic) originates from endometriotic invasion from the outside with unaffected inner components. Subtype III (intramural) resides locally in the myometrium and has no relationship with structural components, and subtype IV (indeterminate) is a heterogeneous mixture of advanced disease (7). The MRI images were shown in Figure 1. For analysis purposes, all the recruited patients were divided into three groups, Group A (extrinsic adenomyosis, subtype II) included recurrent OMA combined with extrinsic adenomyosis, Group B (other subtype adenomyosis) included recurrent OMA combined with other types of adenomyosis (intrinsic adenomyosis, indeterminate adenomyosis, subtype I and subtype IV), and Group C (non-adenomyosis) included patients only with the recurrence of ovarian endometriosis.

General data and clinical features of all patients were retrospectively obtained from searching medical records. The data collected were as follows: age, BMI, menarche age, menstrual cycle, length of menstruations, menorrhagia, obstetrical history (nulligravida, nulliparity), and history of surgery for endometriosis. The clinical features included were: maintenance treatment after the operation (levonorgestrel intrauterine system or long term hormone therapy), the postoperative time to recurrence (TTR ≤ 3, TTR ≤ 5), the recurrence of ovarian cyst and painful symptoms, the occurrence of adenomyosis, the presence and duration of infertility, the painful symptoms (dysmenorrhea, dyspareunia, noncyclic chronic pelvic pain), the intensities of painful symptoms were assessed by a 10-cm visual analog scale (VAS). The intensity of each type of preoperative pain symptom was rated as severe (VAS ≥ 7) or moderate (VAS < 7).

Sonographic features (MRI examination and ultrasound) revealed the presence of pelvic conditions. The size and location of OMA were evaluated in MRI examination. The size of the OMA was defined as the largest diameter of cysts. Except for MRI, uterine size in three dimensions (length, width, and depth) were investigated in ultrasonography. The uterine volume was calculated according to the following formula for ellipsoid bodies: *V* = longitudinal diameter^*^anteroposterior diameter^*^transverse diameter^*^0.523 (16). The elevated serum level of CA125 is defined as >35 U/ml according to the clinical laboratory at our institution.

Surgical procedures were performed mainly *via* laparoscopy (some patients *via* laparotomy). A complete examination of pelvic and abdominal cavity was performed to assess the degree of endometriotic disease; rASRM (revised American Society for Reproductive Medicine) scores were carefully evaluated for staging after surgery. All the patients underwent complete surgical excision of their endometriosis lesion in our institution. In the surgical process, DIE was diagnosed histologically as endometriotic tissue arbitrarily infiltrating beneath the peritoneum surface by >5 mm (17). The DIE sites were classified as five different locations: USL (uterosacral ligaments), the vagina, bladder, intestine, and ureter.

All patients were divided into three groups, subtype II, other types of adenomyosis (subtype I, subtype IV), and nonadenomyosis. Analysis was performed between the three groups to compare the baseline characteristics, clinical features, surgical findings, and sonographic features. IBM SPSS 23.0 software was used for statistical analysis. Data distribution was verified by the Shapiro-Wilk test. Continuous variables were analyzed using Kruskal–Wallis test. Categorical variables were analyzed using Pearson's χ^2^ test or Fisher's exact test. Logistic regression models were used for multivariate analysis, in which the variables included were those found to be statistically significant in the univariate analysis. The odds ratio (OR) and 95% confidence interval (CI) were calculated. All statistical tests were two-sided and differences were considered statistically significant at *p* < 0.05.

## Results

During the work, we recruited 233 patients with recurrence of OMA. We found it was combined with 112 (48.07%, 112/233) subtype II adenomyosis, 8 (3.43%, 8/233) subtype I adenomyosis, 47 (20.17%, 47/233) subtype IV adenomyosis, and 66 (28.32%, 66/233) nonadenomyosis.

The results of comparison of patients' baseline characteristics among the three groups are detailed in Table 1. In recurrent OMA, the women in subtype II group were significantly younger compared with other types of adenomyosis and nonadenomyosis group (38.79 ± 5.21 vs. 39.06 ± 5.49 vs. 40.24 ± 6.21, *p* < 0.05). Moreover, the duration of menstruation in other types of adenomyosis group (especially in internal adenomyosis) was significantly longer (5.80 ± 1.29 vs. 6.51 ± 1.18 vs. 6.43 ± 1.16, *p* < 0.05). The three groups are similar in weight, height, BMI, regular menstrual cycle, mean length of menstruation, heavy menstrual bleeding, nulligravid, nulliparity, history of miscarriages, and previous uterine surgery.

**Table 1:** Baseline characteristics of 233 recurrence of ovarian endometrioma according to the presence of adenomyosis.

	**Subtype II** **(***n*** = 112)**	**Other types of adenomyosis** **(***n*** = 55) -subtype I (8) -subtype IV (47)**	**Non-adenomyosis** **(***n*** = 66)**	***P-*** **value**
Age, y	38.79 ± 5.21	39.06 ± 5.49	40.24 ± 6.21	0.046
Weight, kg	57.35 ± 8.68	55.43 ± 5.97	56.24± 8.94	0.15
Height, cm	159.90 ± 4.97	158.58 ± 4.30	159.72 ± 4.97	0.79
BMI, kg/m2	22.40 ± 3.06	22.05 ± 2.32	22.02 ± 3.16	0.75
Mean age at menarche, y	12.62 ± 1.61	12.82 ± 1.70	12.81 ± 1.64	0.86
Mean duration cycle, d	28.80 ± 3.29	29.51 ± 3.18	29.43 ± 2.96	0.64
Regular menstrual cycle (*n*, %)	99/112 (88.39)	45/55 (81.99)	57/66 (86.36)	0.64
Mean length of menstruations, d	5.80 ± 1.29	6.51 ± 1.18	6.43 ± 1.16	0.58
Heavy menstrual bleeding (*n*, %)	6 (5.36)	5 (9.10)	4 (6.06)	0.042
Nulligravid (*n*, %)	43 (38.39)	13 (23.64)	8 (12.12)	0.08
Nulliparity (*n*, %)	52 (46.43)	21 (38.18)	13 (19.70)	0.07
History of miscarriage (*n*, %)	42(37.5)	25 (45.5)	30 (45.4)	0.33
Previous uterine surgery (*n*, %))	27 (24.11)	18 (32.73)	17 (25.76)	0.57

*Data are presented as means ± the standard deviation and n (%) as appropriate. Kruskal–Wallis followed by post-hoc Dunn's test or ANOVA test; χ^2^-test or Fisher's exact test; p < 0.05*.

The results of comparison of clinical features are detailed in Table 2. Patients receiving postoperative hormonal suppression or LNG-IUD insertion after first conservative OMA surgery were significantly higher in subtype II group (79.46%, 89/112 vs. 63.64%, 35/55 vs. 54.44%, 36/66; 21.43%, 24/112 vs. 14.55%, 8/55 vs. 9.10%, 6/66 respectively, *p* < 0.05). It seems likely that subtype II adenomyosis is more easily combined with recurrence of OMA, and even GnRH and LNG-IUD are more frequently used. The cumulation of 3-years and 5-years recurrence patients are 87 and 182 respectively. In the recurrence of OMA, the rate of subtype II adenomyosis was significantly higher than other adenomyosis and no adenomyosis. The cumulative rate of subtype II adenomyosis in 3-year and 5-year group were 60.92% (53/87) and 46.70% (85/182), respectively. It was suggested that subtype II adenomyosis more easily occurred in early-recurrent patients with endometriosis. Recurrence of OMA combined with adenomyosis was found associated with infertility, especially in subtype II adenomyosis group. The prevalence of no infertility was 50.8% (65/112) in subtype II group, with 78.18% (43/55) in other types of adenomyosis group, and 83.33% (55/66) in the nonadenomyosis group. The infertility rate was significantly higher in subtype II group, which was mainly attributed to primary fertility. The primary infertility rate was significantly higher in subtype II group than other groups (31.25%, 35/112 vs.14.55%, 8/55 vs. 10.77%, 7/66, respectively, *p* = 0.014). The presence of diffuse adenomyosis was not significantly associated with the presence of primary or secondary infertility. The prevalence of pain symptom, duration of pain symptom >5 years, and dysmenorrhea was significantly more severe in subtype II group compared with other groups. Conversely, there is no significant difference in dyspareunia, chronic pelvic pain, CA125 level between subtype II group and other groups.

**Table 2:** Comparison of the clinical features of 233 recurrence of ovarian endometrioma according to the presence of adenomyosis.

	**Subtype II** **(***n*** = 112)**	**Other types of adenomyosis** **(***n*** = 55)**	**Non-adenomyosis** **(***n*** = 66)**	***P-*** **value**
Postoperative GnRH therapy (*n*, %)	89 (79.46)	35 (63.64)	36 (54.55)	0.024
Postoperative LNG-IUD insertion (*n*, %)	24 (21.43)	8 (14.55)	6 (9.10)	0.032
3-year recurrence (n, %)	53/87 (60.92)	16/87(18.39)	18/87 (20.69)	0.028
5-year recurrence (*n*, %)	85/182 (46.70)	56/182 (30.77)	41/182 (22.53)	0.025
Time of OMA occurrence, mon	44.28 ± 8.37	63.96 ± 10.28	69.36 ± 9.34	0.017
Time of adenomyosis occurrence, mon	46.88 ± 12.20	69.76 ± 12.49	-	
Time of pain recurrence, mon	47.12 ± 10.78	48.32 ± 12.08	48.73 ± 12.36	0.56
Pain symptom (*n*, %)	96 (85.71)	38 (69.10)	12 (18.18)	0.013
Duration of pain symptom, mon	28.88 ± 9.02	15.36 ± 7.85	5.67 ± 1.78	0.016
Duration of pain symptom >5 y (*n*, %)	21 (18.76)	7 (12.73)	6 (9.09)	0.67
Dysmenorrhea (*n*, %)	82 (73.21)	30 (54.55)	35 (53.03)	0.027
Dyspareunia (*n*, %)	47 (41.96)	24 (43.63)	25 (37.88)	0.58
Chronic pelvic pain (*n*, %)	27 (24.10)	13 (23.64)	15 (22.73)	0.86
VAS dysmenorrhea	7.81 ± 1.72	5.62 ± 1.61	4.45 ± 1.26	0.034
VAS dyspareunia	4.63 ± 2.81	4.65 ± 2.34	4.34 ± 1.84	0.72
VAS chronic pelvic pain	3.54 ± 2.76	3.64 ± 2.81	3.56 ± 2.17	0.78
Dysmenorrhea sever, VAS ≥ 7 (n, %)	53 (47.32)	26 (47.27)	31 (46.97)	0.69
Dyspareunia severe, VAS ≥ 7 (n, %)	32 (28.57)	17 (30.91)	16 (24.24)	0.83
No infertility (*n*, %)	65 (58.04)	43 (78.18)	55 (83.33)	0.035
Primary infertility (*n*, %)	35 (31.25)	8 (14.55)	7 (10.77)	0.014
Secondary infertility (*n*, %)	12 (10.71)	4 (7.27)	4 (6.06)	0.63
Duration of infertility >5 y (*n*, %)	17 (15.18)	3 (5.45)	2 (3.03)	0.015
Ca125	166.26 ± 28.86	171.2 ± 33.23	100.22 ± 27.64	0.65

*Data are presented as means ± the standard deviation and n (%) as appropriate. Kruskal-Wallis followed by post-hoc Dunn's test or ANOVA test; χ^2^-test or Fisher's exact test; p < 0.05*.

Imaging appearance according to the adenomyosis phenotype is presented in Table 3. Concerning the MRI examination, most of (89.29%, 100/112) the external lesions were in the posterior wall of the uterus. The adenomyosis lesion in subtype II group was larger than other groups. The uterine volume and mean OMA size were significantly larger in subtype II group than other groups. No statistical difference was observed among three groups in myometrium thickness, the presence of leiomyomas, unilateral, or bilateral of endometrioma.

**Table 3:** Imaging appearance of 233 recurrence of ovarian endometrioma according to the presence of adenomyosis.

	**Subtype II** **(***n*** = 112)**	**Other types of adenomyosis** **(***n*** = 55)**	**Non-adenomyosis** **(***n*** = 66)**	***P-*** **value**
Presence of posterior external lesion (*n*, %)	100 (89.29)	30 (54.44)	-	0.021
Size of the posterior external lesion (mm)	38.87 ± 8.17	27.07 ± 4.46	-	0.016
Presence of the anterior external lesion (*n*, %)	12 (10.71)	25(45.46)	-	0.018
Size of the anterior external lesion (mm)	21.14 ± 5.53	18.89 ± 4.36	-	0.029
JZ zone thickness, mm	7.46 ± 4.83	12.25 ± 2.62	5.47 ± 1.21	0.022
Myometrium thickness, mm	15.77 ± 5.61	16.79 ± 5.94	14.82 ± 4.18	0.46
Junctional zone/myometrium ratio	0.46 ± 0.22	0.78 ± 0.35	0.37 ± 0.16	<0.01
Presence of leiomyomas (*n*, %)	15 (13.39)	8 (14.55)	9 (13.64)	0.77
Uterine volume, cm^3^	257.37 ± 42.61	203.14 ± 33.52	100.85 ± 26.67	<0.01
Presence of endometrioma (*n*, %)				
Bilateral	63 (56.25)	28 (50.90)	19 (28.79)	0.22
Unilateral	49 (43.75)	27 (49.10)	47 (71.21)	0.37
Right	20	17	20	
Center	29	20	27	
Mean OMA size, cm	4.97 ± 2.25	4.36 ± 2.38	4.46 ± 2.70	0.031
Right	4.49 ± 2.21	3.91 ± 2.81	4.71 ± 2.62	0.042
Center	5.01 ± 2.13	4.79 ± 2.34	4.22 ± 2.46	0.029

*Data are presented as means ± the standard deviation and n (%) as appropriate. Kruskal–Wallis followed by post-hoc Dunn's test or ANOVA test; χ^2^-test or Fisher's exact test; p < 0.05*.

As for the surgical procedure (Table 4), the rate of DIE occurrence was significantly higher in the subtype II group than other groups (94/112, 83.93% vs.25/55, 45.45% vs. 27/66, 40.91%). The mean total number of DIE lesion was significantly higher in subtype II group compared with other groups (3.6 ± 1.8 vs. 2.3 ± 1.5 vs. 2.2 ± 1.3, respectively, *p* < 0.01). In the recurrence of ovarian endometriosis, the subtype II patients exhibited more significantly associated with DIE. Twenty-one patients had partial and 91 patients had total cul-de-sac obliteration in subtype II group. According to the rASRM classification, 83 (74.10%) cases had stage III-IV endometriosis in subtype II group, 28 (50.91%) cases had stage III-IV endometriosis in other types of adenomyosis group, 26 cases had stage III–IV endometriosis in nonadenomyosis. The prevalence of stage III–IV endometriosis in extrinsic adenomyosis group was the highest among the three groups.

**Table 4:** Comparison of the surgical findings of 233 recurrence of ovarian endometrioma according to the presence of adenomyosis.

	**Subtype II** **(***n*** = 112)**	**Other types of adenomyosis** **(***n*** = 55)**	**No adenomyosis** **(***n*** = 66)**	***P-*** **value**
DIE rate (*n*, %)	94 (83.93)	25 (45.45)	27 (40.91)	0.014
Mean total no. Of die lesions	3.6 ± 1.8	2.3 ± 1.5	2.2 ± 1.3	<0.01
No. Of lesions *n* = 1 (*n*, %)	31 (27.68)	27 (49.10)	38 (57.58)	
No. Of lesions n ≥ 2	81 (72.32)	28 (50.91)	28 (42.42)	<0.01
Usl	87 (77.68)	32 (58.18)	14 (21.21)	<0.01
Bilateral	63 (72.41)	12 (37.5)	6 (42,86)	
Unliteral	24 (27.59)	20 (62.5)	8 (57.14)	
Right	11	9	4	
Left	13	10	4	
Ureter	45 (40.18)	14 (25.45)	6 (9.10)	0.03
Bilateral	32 (71.11)	8 (57.14)	3 (50.00)	
Unliteral	13 (28.89)	6 (42.86)	3 (50.00)	
Right	6	3	2	
Left	7	3	1	
Vagina	32 (28.57)	12 (21.82)	0	<0.01
Bladder	4 (3.57)	0	0	<0.01
Intestine	13 (11.61)	5 (9.10)	2 (3.03)	<0.01
Punch of Douglas				
Partial	21 (18.75)	25 (45.45)	36 (54.55)	<0.01
Total	91 (81.25)	30 (54.55)	30 (45.45)	<0.01
Mean total rasrm score	103.14 ± 23.89	74.23 ± 16.72	36.51 ± 14.23	<0.01
Mean implant rasrm score	32.66 ± 12.37	26.57 ± 13.45	24.45 ± 12.34	0.023
Mean adhesion rasrm score	36.71 ± 14.57	17.65 ± 10.42	15.63 ± 10.26	0.017
Stage III/IV	83 (74.10)	28 (50.91)	26 (39.40)	<0.01

*Data are presented as means ± the standard deviation and n (%) as appropriate. Kruskal–Wallis followed by post-hoc Dunn's test or ANOVA test; χ^2^-test or Fisher's exact test; p < 0.05*.

The study compared recurrent OMA combined with subtype II and other types of adenomyosis, nonadenomyosis in terms of their general baseline characteristics, clinical features, imaging appearance, and surgery findings. Then we tried to define whether a preoperative diagnose on extrinsic adenomyosis would help to predict the recurrence of OMA. This allowed us to determine whether the extrinsic adenomyosis was associated with the early recurrence of OMA (Table 5). We performed a univariate analysis comparing the general clinical characteristics according to the presence of early recurrence of OMA (in 3 years) or late recurrence of OMA(>3 years); next we created a multiple logistic regression analysis to investigate the risk factors of early recurrence of OMA. Variables significantly associated with early OMA recurrence in the univariate analysis were pain symptom, VAS of pain symptom, extrinsic adenomyosis, DIE, primary infertility, extrinsic adenomyosis with DIE, and extrinsic adenomyosis with primary infertility. The extrinsic adenomyosis (OR 2.5, 95% CI 1.2–3.4), DIE (OR 2.1, 95% CI 1.4–2.8), and primary infertility (OR 1.8, 95% CI 1.3–4.3) were significantly and independently associated with early recurrence of OMA, especially extrinsic adenomyosis with DIE or primary infertility.

**Table 5:** Risk factors of early recurrence (in 3-year) of ovarian endometrioma in univariate and multivariate analysis.

	**≤ 3 years** **(***n*** = 87)**	**>3 years** **(***n*** = 146)**	**Univariate analysis** ***p*****-value**	**Multivariate analysis OR (95%CI)**	***p*** **-value**
Age, y	35.26 ± 3.26	36.17± 4.18	0.38		
BMI, kg/m2	22.78 ± 3.44	23.14 ± 3.08	0.23		
Nullgravidy (*n*, %)	28 (32.18)	47 (32.19)	0.61		
Nullparity (n, %)	32 (36.78)	52 (35.62)	0.54		
Postoperative GnRH therapy (*n*, %)	42 (48.28)	72 (49.32)	0.42		
Postoperative LNG-IUD insertion (*n*, %)	45 (51.72)	75 (51.37)	0.71		
Endometrioma surgery history (*n*, %)	17 (19.54)	30 (20.55)	0.29		
Leiomyoma (*n*, %)	17 (19.54)	32 (21.92)	0.77		
Pain symptom (*n*, %)	61 (70.11)	65 (44.52)	0.024	1.6 (0.8–2.3)	0.09
VAS of pain symptom	7.28 ± 1.72	5.76 ± 1.29	0.029	1.5 (0.7–2.7)	0.21
Extrinsic adenomyosis (*n*, %)	53 (60.92)	59 (40.41)	<0.01	2.5 (1.2–3.4)	<0.01
DIE (*n*, %)	69 (79.31)	77 (52.74)	0.018	2.1 (1.4–2.8)	0.012
Primary infertility (*n*, %)	32 (36.78)	18 (12.32)	0.012	1.8 (1.3–4.3)	0.011
Secondary infertility (*n*, %)	7 (8.05)	13 (8.90)	0.56	2.2 (0.6–3.8)	0.37
Extrinsic adenomyosis with DIE (*n*, %)	42 (48.28)	26 (17.81)	<0.01	3.8 (2.1–5.6)	<0.01
Extrinsic adenomyosis with primary infertility (*n*, %)	28 (32.18)	10 (6.85)	<0.01	3.2 (2.2–5.1)	<0.01

*OR, odds ratio, CI, confidence interval, a multiple logistic regression analysis. p < 0.05*.

## Discussion

Both endometriosis and adenomyosis are defined as the presence of endometrial glands and stroma outside of the uterine and within the uterine myometrium, respectively (18). These diseases are characterized with estrogen dependence (19). OMA is the most common type, and the occurrence rate of endometriosis is rising in recent years (20). Rate of endometriosis recurrence varies from 9 to 60% in different studies, depending on the definition of “recurrence.” Recurrence is variously defined based on different aspects, such as radiographic evidence of endometriotic lesion (ultrasound [US] or magnetic resonance image [MRI]), recurrence of clinical symptoms including pelvic pain (dysmenorrhea, dyspareunia, or noncyclic pelvic pain) measured using the visual analog scale (VAS), or as a rise of CA125 level after surgery. Some risk factors have been found involved in the consequence of recurrence, such as younger age, high body mass index (BMI), and no-complete surgical excision (21).

Previous study confirmed that adenomyosis is an independent risk factor for the recurrence of endometriosis after surgery during the long-time follow up (5). Both endometriosis and adenomyosis have adverse clinical symptoms, especially in infertility (22). In this study, we set out to assess the correlation between extrinsic adenomyosis and recurrence of OMA for the first time. One interesting finding is that the rate of extrinsic adenomyosis combined with recurrent OMA was up to 48.07%. Extrinsic adenomyosis is closely related to the recurrence of OMA. We observed that extrinsic adenomyosis often shows more serious pain symptoms and infertility. Compared with other groups, extrinsic adenomyosis was often accompanied by larger cysts and uterine volume. In addition, extrinsic adenomyosis is associated with more severe DIE lesions and higher ASRM scores. Another important finding is that extrinsic adenomyosis, DIE lesions, and primary infertility were significantly associated with early recurrence (in 3-year) of OMA.

Although several studies have investigated adenomyosis symptoms (7), few have compared the clinical profiles according to adenomyosis combined by recurrent endometriosis. In our work, we found that the patients age was significantly younger in extrinsic adenomyosis combined recurrent endometriosis. Conversely, the patients in other adenomyosis group showed a marked increase of length of menstruation (23). Several works reported in the literature have presented findings consistent with our results. In a work comparing intrinsic adenomyosis versus extrinsic adenomyosis in 248 women diagnosed by MRI, the women with external adenomyosis were significantly younger and more likely to exhibit an associated endometriosis (23). A higher rate of heavy menstrual bleeding and a longer duration of menstruation were found in the internal adenomyosis group compared with the external adenomyosis group, which is in accordance with our results.

Clinically, diagnosing the adenomyosis was based on the patients' symptom, especially in dysmenorrhea (24). Meanwhile, some patients were asymptomatic resulting in a clinically neglected condition and lack of specificity which makes the diagnosis delayed (25). In our work, we found that the time of adenomyosis occurrence is late after the time of OMA recurrence. Subtype II group exhibited adenomyosis lesion larger than other groups. The VAS score of dysmenorrhea was higher in subtype II group compared with other groups. A previous study reported that the dysmenorrhea severity is associated with the depth and degree of invasion of adenomyosis into the myometrium (26).

The prevalence of infertility in adenomyosis is still insufficiently elucidated in reproductive women (27). We found that the infertility rate was significantly higher in extrinsic adenomyosis group than other types of adenomyosis group. Primary infertility exhibited a strong association with subtype II group, and other types of adenomyosis did not appear to be associated with fertility status (28). In a study, 496 women between 18 and 42 years of age were divided into three groups according to fertility condition, and the rate of focal extrinsic adenomyosis was significantly increased in the primary infertility group (29). Meanwhile, Li et al. reported that adenomyosis with larger uterine volume might have a higher incidence of miscarriage (30). Our results, in addition to previous data, have confirmed the potential role of adenomyosis in primary infertility.

Endometriosis and adenomyosis shared many common similarities, such as dysmenorrhea, dyspareunia, chronic pelvic pain and infertility. Chapron et al. have already shown that focal extrinsic adenomyosis occurs more frequently in endometriotic patients and that it is significantly correlated with the DIE endometriosis phenotype (10). Recently, Chapron et al. also found that focal adenomyosis of the outer myometrium (FAOM) is associated with greater DIE severity and higher rASRM score, in addition, coexisting focal extrinsic adenomyosis and DIE was associated with more severe DIE lesions. FAOM refers exclusively to subtype II of the originally described Kishi' classification (31). In our work, we have already confirmed that subtype II adenomyosis was observed more frequently in recurrent endometriosis, and subtype II adenomyosis was significantly and independently associated with DIE lesions. In our work, adenomyosis lesions in subtype II (89.29%, 100/112) more easily occurred in the posterior of wall of the uterus. On the other hand, a study done in 39 women with histologically proven bladder DIE and preoperative MRI examination showed 50% women with bladder DIE had focal adenomyosis of the anterior wall of the uterus (32). Therefore, our data confirmed that there is a close connection between extrinsic adenomyosis and DIE, which shared the same pathogenic pathway and consequence of the diseases (33).

In our work, we also found that patients in subtype II group were with larger uterus volume and diameter of recurrent cysts. This supports the notion that ectopic endometriotic cells in the pelvic could be the progenitor of extrinsic adenomyosis (34). Previous studies have reported that adenomyosis and endometriosis represent different phenotypes of a single disease. The coexistence of adenomyosis and DIE was not a rare occurrence (35). Sampson divided adenomyosis into 3 groups according to the origin or pathogenesis: invasion from within the uterus (intrinsic adenomyosis); invasion from outside the uterus (extrinsic adenomyosis); and misplaced endometrial tissue in the uterine wall (diffuse adenomyosis) (36). This theory led to Kishi's classification criteria (7). Ectopic endometriotic cells in the pelvic infiltrated the peritoneum, the ureters, the bladder and then invaded the rectum and the outer part of the uterus, triggering adhesion that furthermore promoted creating posterior cul-de-sac obliteration and disrupting the uterine serosa to create extrinsic adenomyosis (37). Deep endometriosis that is located within Cul-de-sac is also called adenomyotic lesions, thus including deep endometriosis, extrinsic adenomyosis, and uterine enlargement (38). This suggests that extrinsic adenomyosis might originate from the invasion of adjacent DIE lesions (31).

Uterine volume is considered as a monitoring indicator of disease condition in adenomyosis patients. Uterine enlargement is associated with increased miscarriage rate. Li reported that live birth rate is significantly lower in adenomyosis patients with an enlarged uterus undergoing frozen-thawed embryo transfer (30). Adenomyosis is related to stage III–IV Endometriosis and higher rate of endometriosis recurrence (14). Monitoring uterine volume should be taken into the postoperative management of endometriosis patients. The surgeon should pay attention to the uterine volume, especially in extrinsic adenomyosis. It will benefit patients to prevent the recurrence of endometriosis and improve pregnancy outcome.

The strength of this study is based on the following aspects. This is the first study that has identified the clinical features, imaging appearance, surgery findings of adenomyosis combined with the recurrence of OMA. The selection of patients with recurrent endometriosis was based on strict surgical and histological criteria. All the study patients underwent a preoperative pelvic MRI with a high level of expertise in gynecological imaging. The diagnosis of adenomyosis was based on strict MRI criteria. Clinical data and surgery information were recorded fully and accurately, prospectively. Our result added a piece of evidence to the theory that there is an overlap in the pathogenesis of endometriosis and adenomyosis (15). Our study also has some limitations. This study was performed in a population of patients who required surgical intervention of recurrent OMA, assuming that asymptomatic patients were not scheduled for reexamination and were not included in the study. This could affect the external validity of the study. The study only included the women with the recurrence of ovarian endometriosis, and recurrent DIE patients without OMA was not included. It is unclear whether extrinsic adenomyosis is a risk factor of DIE.

Our study showed baseline characteristics, clinical features, imaging appearance, and surgery findings between extrinsic adenomyosis and other types of adenomyosis. We found that extrinsic adenomyosis is more frequently observed in recurrent OMA. Extrinsic adenomyosis often showed more adverse symptoms. Extrinsic adenomyosis is associated with more severe DIE lesions and higher ASRM scores. It will be beneficial for patients of coexistence of adenomyosis and endometriosis to apply individual management to achieve better efficacy (39). The coexistence of adenomyosis and endometriosis must be part of the decision-making process for DIE patients when they are presented on abnormal clinical symptoms and long-term infertility (40). Furthermore, our study confirmed a strong link between extrinsic adenomyosis and pelvic endometriosis that share a similar pathophysiological basis. This could be an important turning point in diagnosis and treatment of these two diseases. Further prospective studies are required to develop positive effects to help with management and prognosis of endometriosis and adenomyosis.

## Conclusion

Our study presented that extrinsic adenomyosis is closely related to postoperative recurrence of OMA. We observed that extrinsic adenomyosis often shows more serious pain symptoms and infertility, and it was often accompanied by larger cysts and uterine volume. In addition, extrinsic adenomyosis is associated with more severe DIE lesions and higher ASRM scores. Extrinsic adenomyosis, DIE lesions, and primary infertility were significantly and independently associated with early recurrence of OMA. Our data confirmed there is a strong link between extrinsic adenomyosis and endometriosis. Furthermore, the coexistence of adenomyosis and endometriosis needs a decision-making process based on abnormal clinical symptoms and infertility condition in practice.

## Data Availability Statement

The original contributions presented in the study are included in the article/supplementary material, further inquiries can be directed to the corresponding author.

## Ethics Statement

The studies involving human participants were reviewed and approved by Women's Hospital, Zhejiang University School of Medicine, Hangzhou, China. The patients/participants provided their written informed consent to participate in this study.

## Author Contributions

XZ developed the idea for the project. The study was designed by MS and PX performed the data analysis and taken full responsibility for the integrity of the data. GZ, JW, and LZ supervised and reviewed the statistical analyses. All authors approved the final version.

## Funding

This study was funded by the National Key R&D Program of China (Grant Number SQ2017YESF080001) and the National Natural Science Foundation of China (Grant Numbers 81974225, 82171636).

## Conflict of Interest

The authors declare that the research was conducted in the absence of any commercial or financial relationships that could be construed as a potential conflict of interest.

## Publisher's Note

All claims expressed in this article are solely those of the authors and do not necessarily represent those of their affiliated organizations, or those of the publisher, the editors and the reviewers. Any product that may be evaluated in this article, or claim that may be made by its manufacturer, is not guaranteed or endorsed by the publisher.
